# Correction to: sciCNV: high-throughput paired profiling of transcriptomes and DNA copy number variations at single-cell resolution

**DOI:** 10.1093/bib/bbae009

**Published:** 2024-01-10

This is a correction to: Ali Mahdipour-Shirayeh, Natalie Erdmann, Chungyee Leung-Hagesteijn, Rodger E Tiedemann, sciCNV: high-throughput paired profiling of transcriptomes and DNA copy number variations at single-cell resolution, *Briefings in Bioinformatics*, Volume 23, Issue 1, January 2022, bbab413, https://doi.org/10.1093/bib/bbab413

In the originally published version of this manuscript, author Ines Tagoug was inadvertently omitted from the author list.

The author list, the authors' biographical information, and the authors' contributions should have been given as follows:

Ali Mahdipour-Shirayeh, Ines Tagoug, Natalie Erdmann, Chungyee Leung-Hagesteijn and Rodger E. Tiedemann^†^

Corresponding author. Rodger E. Tiedemann, Princess Margaret Cancer Centre, University Health Network, Toronto, Ontario, Canada; Department of Medicine, University of Toronto, Toronto, Ontario, Canada; E-mail: rodger.tiedemann@uhn.ca

^†^Rodger E. Tiedemann is a senior author.

**Ali Mahdipour-Shirayeh** is a Research Associate and data scientist at the Princess Margaret Cancer Centre, University Health Network, Toronto, Canada

and the Faculty of Medicine, University of Toronto, Toronto, Canada. He received his PhD in Biomedical Science from University of Waterloo, Canada for studies related to the evolution of cancer and the analysis of genetic data. His research interests include data science, machine learning, AI, computational biology and cancer evolution.

**Ines Tagoug** is a Scientific Associate at the Princess Margaret Cancer Centre, University Health Network, Canada. She received her PhD in Cancer Research from University Claud Bernard, Lyon, France. Her research interests include immunotherapy and intra-tumor heterogeneity using sequencing and bioinformatics.

**Natalie Erdmann** is a senior research technician and laboratory manager at the Princess Margaret Cancer Centre, University Health Network, Canada. She has a M.Sc. in Molecular and Medical Genetics from the University of Toronto and is currently engaged in studying the genetics of multiple myeloma in primary patient samples.

**Chungyee Leung-Hagestejin** has a M.Sc. in Microbiology and Immunology from McGill University, Montreal, and worked as a senior research technician and lab manager for 35 years at Princess Margaret Cancer Centre, University Health Network, Canada. She has expertise in cell/molecular biology, immunology and cancer biology.

**Rodger E. Tiedemann** is a Senior Scientist at The Princess Margaret Cancer Centre and is Associate Professor of Medicine and Medical Biophysics at the University of Toronto, in Toronto, Ontario, Canada. He was a national representative to the 29th International Mathematics Olympiad and completed PhD and medical degrees at the University of Auckland in Auckland, New Zealand. His research interests include intra-tumor heterogeneity and immunotherapy in multiple myeloma, single cell genomics and bioinformatics.

**Authors' contributions** 

A.M.-S. performed research and analyzed data. N.E., C.L.-H. and I.T. took care of FISH, FACS and WES, respectively. R.E.T. designed research, analyzed the data and wrote the paper.

These details have been corrected only in this correction notice to preserve the published version of record.

